# The Effects of Different Space Forms in Residential Areas on Outdoor Thermal Comfort in Severe Cold Regions of China

**DOI:** 10.3390/ijerph16203960

**Published:** 2019-10-17

**Authors:** Zheming Liu, Yumeng Jin, Hong Jin

**Affiliations:** School of Architecture, Harbin Institute of Technology; Key Laboratory of Cold Region Urban and Rural Human Settlement Environment Science and Technology, Ministry of Industry and Information Technology, Harbin 150006, China; 14b334013@hit.edu.cn (Z.L.); 17b334004@stu.hit.edu.cn (Y.J.)

**Keywords:** outdoor thermal comfort, thermal environment, forms and configurations of the spaces, field measurement, residential areas, severe cold regions

## Abstract

In the context of global climate change and accelerated urbanization, the deterioration of the urban living environment has had a serious negative impact on the life of residents. However, studies on the effects of forms and configurations of outdoor spaces in residential areas on the outdoor thermal environment based on the particularity of climate in severe cold regions are very limited. Through field measurements of the thermal environment at the pedestrian level in the outdoor space of residential areas in three seasons (summer, the transition season and winter) in Harbin, China, this study explored the effects of forms and configurations of three typical outdoor spaces (the linear block, the enclosed block, and the square) on the thermal environment and thermal comfort using the Physiologically Equivalent Temperature (PET). The results show that the thermal environment of all outdoor space forms was relatively comfortable in the transition season but was uncomfortable in summer and winter. The full-enclosed block with a lower sky view factor (SVF) had a higher thermal comfort condition in summer and winter. The linear block with higher buildings and wider south–north spacing had a higher thermal comfort condition in summer and winter. When the buildings on the south side were lower and the south–north spacing was wider, the thermal environment of the square was more comfortable in winter.

## 1. Introduction

Global climate change is one of the major environmental problems mankind faces in the 21^st^ century [1]. The World Health Organization (WHO) has estimated that climate change could cause 250,000 additional deaths per year between 2030 and 2050 [2]. In the context of global climate change and accelerated urbanization, much attention has been paid to the issue of the urban thermal environment [3,4,5]. As the basic unit of the city, the residential area is most closely related to people’s living, so its thermal environment has a direct impact on residents’ physical and mental health and their thermal comfort during outdoor activities [6]. Therefore, in the early stage of planning, the effects of the spatial forms and configurations of the residential areas on the outdoor thermal environment should be fully considered so that a comfortable outdoor activity space and residential environment can be created [7,8,9,10].

In order to assess the outdoor thermal comfort, thermal indices, including PET [11], Predicted Mean Vote (PMV) [12], Universal Thermal Climate Index (UTCI) [13], Standard effective temperature (SET*) [14], Temperature-Humidity Index (THI) [15,16], Discomfort Index (DI) [17], and Wet-Bulb Globe Temperature (WBGT) [18], are widely used. Among them, THI, DI and WBGT are empirical indices derived from subjective comfort estimates, whereas PET, PMV, UTCI and SET* are rational indices based on the heat balance equation of the human body [19]. PMV and SET* are typically applied for a relatively stable indoor environment, while PET and UTCI have been primarily designed for outdoor environment [20]. Moreover, in recent studies on outdoor thermal comfort, PET is the most broadly uesd index [21].

Currently, scholars have already widely studied the effects of different forms and configurations of urban spaces (more focus on urban canyons, courtyards, and squares) on the outdoor thermal environment and thermal comfort and the corresponding results have been acquired.

Johansson et al. [22] made long-term observations on the thermal environment of two streets with different height to width ratios (H/W) in Morocco and analyzed the thermal comfort with PET. The results indicated that the deeper canyon was more comfortable in summer, whereas the shallower canyon was more comfortable in winter due to incoming solar radiation. Krüger et al. [23] conducted field measurements of the microclimate in streets and squares in Brazil, created a thermal sensation equation based on the questionnaire survey and demonstrated the relationship between the SVF and the daytime thermal comfort of pedestrians. Ali-Toudert and Mayer [24,25] analyzed the effects of street canyon geometry on the outdoor thermal environment and thermal comfort in summer through field measurement and simulation. The results indicated that direct solar radiation had a significant effect on the thermal comfort of the human body; as the H/W ratio increases, the temperature slightly decreases. Meanwhile, when the orientation of the street tends to be parallel to the inflow wind direction, there is a reduction in the PET.

Martinelli and Matzarakis [26] studied the effects of the H/W ratio and the SVF on thermal comfort in Italian courtyards, and the results indicated that the SVF has a great effect on the incoming solar radiation in the courtyard, which was more significant in summer. In addition, the increase of the H/W ratio can effectively improve the outdoor thermal comfort of the courtyard in winter and summer. Meir et al. [27] conducted field measurements of the air temperature in two semi-enclosed courtyards with different orientations in Israel and the results indicated that overheat occurred during daytime in summer in the courtyards, regardless of their orientation. However, if the orientation of the courtyard is fully considered based on the solar angle and wind direction, the thermal environment of the space can be improved. Nasrollahi et al. [28] simulated the thermal performance of traditionally designed courtyards with different orientations and H/W ratios in Shiraz, Iran. They showed that the traditional courtyard with a high H/W ratio and southward orientation could obtain better shading in summer as well as allowing the sun’s rays in while regulating the wind speed in winter. Jin et al. [29] studied the microclimate of Chinese–Baroque historic conservation areas in Harbin, China through field measurements and simulation. They showed that the rectangular courtyard had better windbreak performance compared with the T-shaped and L-shaped courtyard; the squares had a good performance in cold resistance, and the higher the degree of enclosure of the square, the lower the internal wind speed.

Taleghani et al. [30] studied the effects of three main urban forms (singular form, linear form and courtyard form) on outdoor thermal comfort in Holland by simulation, and the results indicated that the courtyard form had higher thermal comfort conditions, whereas the singular form had the lowest one due to the long exposure to direct solar radiation. Thorsson et al. [31] in a simulation study of the effects of urban geometry on outdoor thermal environment in Gothenburg, Sweden found out that the squares were warmer than narrow street canyons in summer, but cooler in winter. They also showed that the densely built structure mitigated extreme swings in mean radiation temperature and PET, improving outdoor comfort both in summer and in winter. Yezioro et al. [32] studied the effects of the length to width ratio (L/W), the height of buildings around and the orientation on solar radiation and the results indicated that for regions of latitudes 26–34°, the best orientations for squares were N–S, NW–SE, and NE–SW, and solar radiation can be increased by improving the L/W in those orientations, but the buildings around should not be taller than half the width of the square. Lin et al. [33] conducted field measurements of the microclimate of streets and courtyards in Harbin, China. They showed that the temperature of the Northeast-Southwest oriented street was higher than that of the Northwest-Southeast oriented street in winter and summer; the courtyards with higher SVF had higher temperatures in summer and had higher wind speeds in winter and summer.

Currently, the effects of forms and configurations of urban spaces under the conditions of the hot dry climate and the hot humid climate on the outdoor thermal environment and thermal comfort have been extensively studied, whereas relatively few studies have been conducted on severe cold regions, with a lack of comprehensive consideration of the outdoor thermal environment in different seasons. This study aimed to explore the effects of forms and configurations of outdoor spaces in residential areas on the thermal environment and the thermal comfort in severe cold regions of China. Through field measurements in three seasons (summer, the transition season and winter), the study analyzed the effects of forms and configurations of three typical outdoor spaces (the linear block, the enclosed block, and the square) on air temperature (T_a_), mean radiation temperature (T_mrt_) and wind speed (V_a_). The assessment of thermal comfort was based on PET, calculated using the RayMan software. As the SVF were in inverse proportion to the H/W ratio in the linear block and enclosed block, the SVF, building spacing, building height, etc., were selected as the parameters of configurations to study the effects on the thermal environment. This study provides a reference and evaluation basis for the layout planning of residential areas of severe cold regions.

## 2. Methods

### 2.1. Field Measurement Area

**Figure 1:**
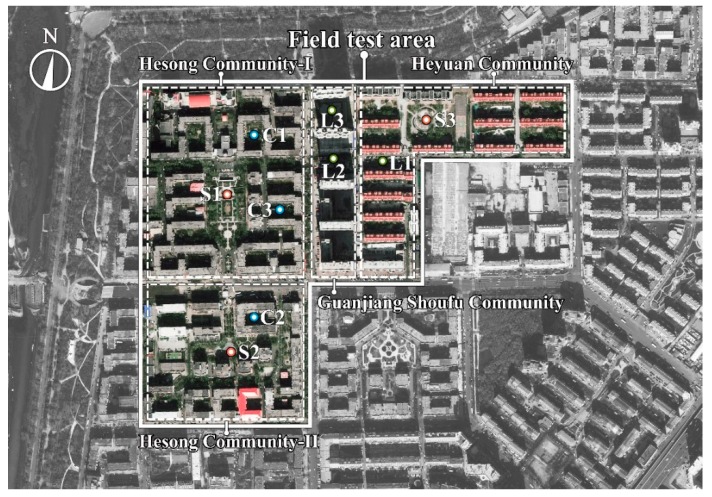
Field measurement site, locations of the selected residential areas and measurement points.

Harbin is a city with 9.5 million inhabitants in Northern China, located between eastern longitude 125°42′–130°10′ and northern latitude 44°04′–46°40′, with an altitude of 142 m, in a region of mid-temperate continental monsoon climate with a cold dry winter season [34]. In Harbin, the linear block, the enclosed block and the square are the most common outdoor space forms in residential areas [35,36]. This study chose four residential areas as the study object: Hesong Community-I, Hesong Community-II, Heyuan Community, and Guangjiang Shoufu Community, which are all located in the central area of the city and close to each other, as shown in Figure 1. The four residential areas also present the representative features of the residential areas in Harbin: (1) They contain the most common outdoor space forms; (2) The main orientation of the buildings is 10° north by east; (3) The building façade is decorated with light color coating, and the materials of the underlying surface are mainly concrete and cement bricks.

**Figure 2:**
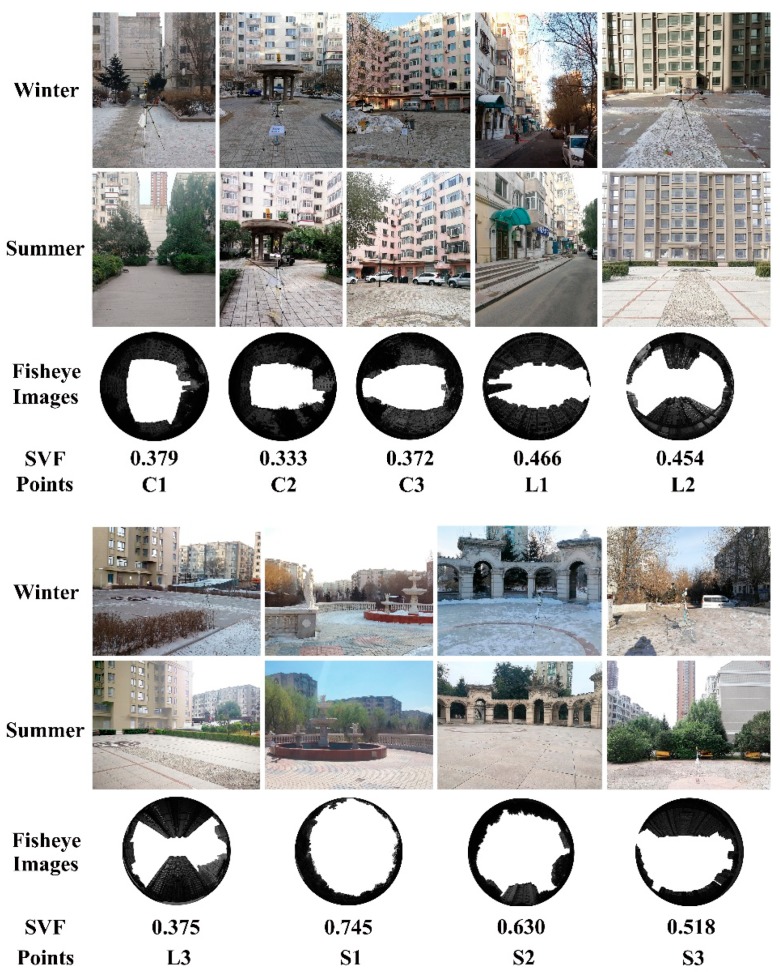
Site environments (winter and summer), fisheye images and SVF.

All the measurement points were located on cement bricks, far from large grasslands, trees and shrubs to avoid their effects. Figure 2 shows the site environment, the fisheye images and the SVF. SVF refers to the ratio of the visible sky that can be seen from a point to the total possible sky hemisphere and can be acquired through the calculation of fisheye images with the RayMan software [37,38]. The fisheye images in Figure 2 were all shot on measured days in summer, as the trees blocked the visible area of the sky very little, the same SVF of each point was used for different seasons.

**Figure 3:**
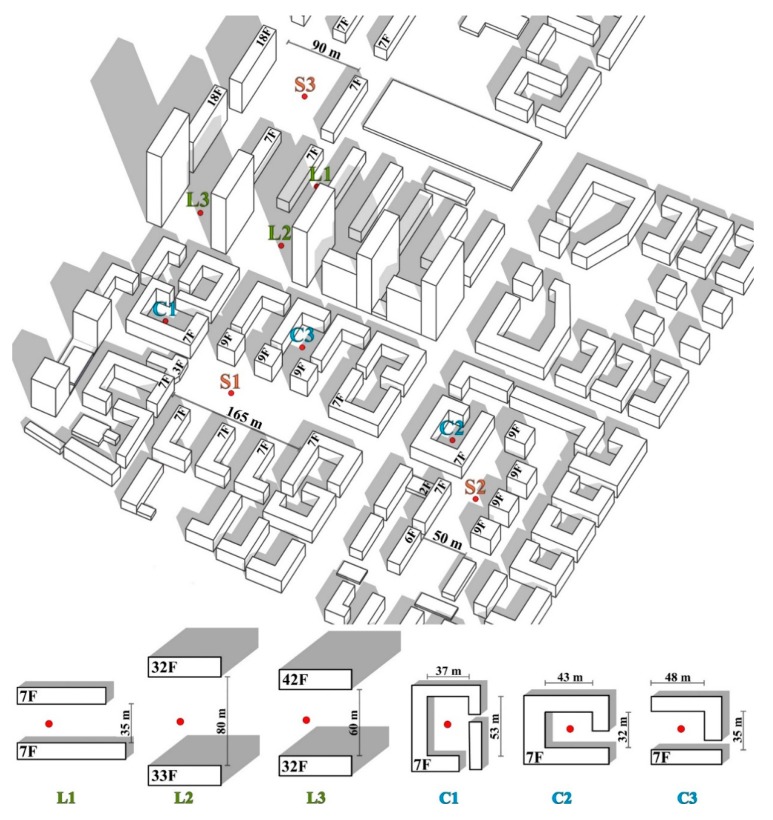
The configurations of the outdoor space at each measurement point.

Figure 3 shows the configuration of the outdoor space at each measurement point. Points C1–C3 were set in the middle of the enclosed block, in which C1 and C2 were in the full-enclosed block, and C3 was in the semi-enclosed block. Points L1–L3 were set in the middle of the linear block and Points S1–S3 were set in the middle of the squares.

### 2.2. Measurement Instruments

As thermal comfort is an important way to assess outdoor thermal environment and the meteorological parameters that mainly affect it include T_a_, V_a_, relative humidity (RH) and T_mrt_ [30]. In this study, fixed-point measurements of the T_a_, RH, V_a_, and globe temperature (T_g_) were carried out at the pedestrian level, and T_mrt_ was acquired through the calculation of the measurement results of the above meteorological parameters.

**Figure 4:**
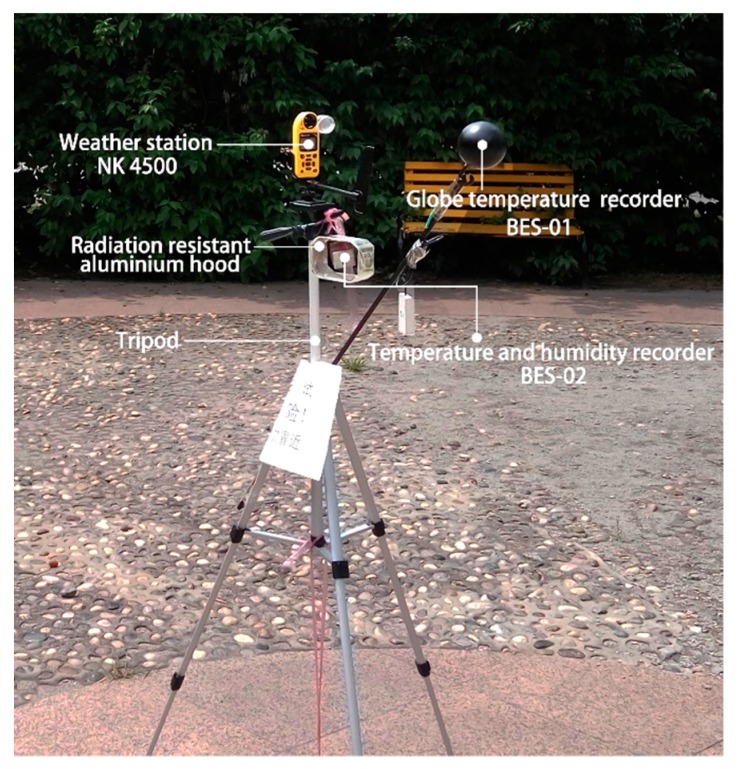
Photograph of the measurement instruments.

Table 1 lists the specifications of the instruments used to measure the meteorological parameters. All the instruments complied with ISO7726 [39]. As shown in Figure 4, the weather station, the globe temperature recorder and the temperature and humidity recorder were fixed at 1.5 m above the ground with tripods; the temperature and humidity recorder was placed inside a radiation-resistant aluminum hood in order to avoid the effects of solar radiation on the measurement results, the ends of the hood were open and well ventilated. The interval of automatic data recording of all the instruments was 1 min.

### 2.3. Weather Conditions

Harbin, a city of China, is located in severe cold regions which are defined as the average T_a_ is not higher than −10 °C in the coldest month, and the number of the day below average T_a_ of 5 °C is not less than 145 days [40].

According to the meteorological data of Harbin in the last two decades (1999–2018), the annual average T_a_ is 5.2 °C, with an average highest T_a_ of 10.5 °C and an average lowest T_a_ of 0.1 °C, and the average V_a_ is in the range of 2.1–3.3 m/s. Moreover, in winter the monthly average T_a_ varies within the range of −22.9–−7.0 °C and the monthly average RH ranges 50.5–80.7% (Dec–Feb), and January is the coldest month, with the daily average T_a_ ranging from −28.2 °C to −5.8 °C. In spring (the transition season) the monthly average T_a_ is in the range of −7.4–18.4 °C and the monthly average RH ranges 35.7–70.5% (Mar–May), and April is the most typical month, with the daily average T_a_ ranging between −0.7 and 17.7 °C. In summer the monthly average T_a_ and RH are in the ranges of 18.9–25.5 °C and 45.5–83.2% respectively (June–August), and July is the hottest month, with the daily average T_a_ ranging 18.2–29.7 °C [41,42].

**Figure 5:**
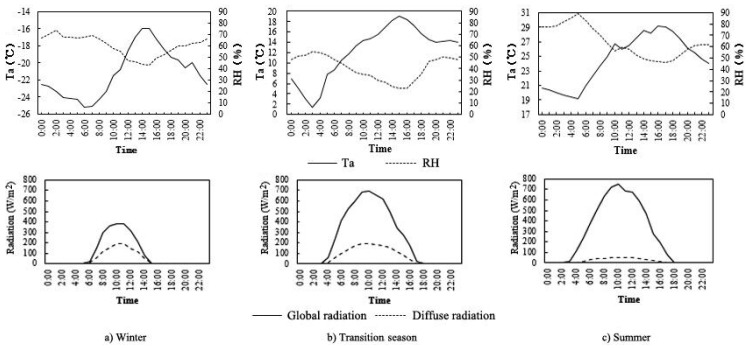
Diurnal variation of T_a_, RH, and solar radiation of the measured days in different seasons.

This study conducted field measurements on 18 July 2016, 28 April 2016 and 11 January 2016, respectively. The meteorological data of the three measured days were from the meteorological observatory of Harbin [42,43]. The average T_a_, the average RH, the average and maximum V_a_ and the prevailing wind direction of the measured days in different seasons are shown in Table 2. The climatic characteristics of the three measured days were consistent with that of the three seasons, so the data of field measurements can represent the microclimate status of the corresponding season. The curves of diurnal variation of Ta, RH, and solar radiation are shown in Figure 5.

### 2.4. Thermal Comfort Indices

T_mrt_ is defined as the uniform surrounding temperature in an imaginary enclosure in which the radiant heat transfer from a human body to the enclosure surfaces is equal to the heat transfer to the surfaces of an actual enclosure with non-uniform temperatures. T_mrt_ is a key input variable to calculate thermal comfort indices, being directly related to solar radiation [23,44]. T_mrt_ is calculated according to the forced convection of the ISO7726 standard [39], expressed as:

(1) $$T_{\mathrm{mrt}}={\left[{\left(T_{g}+273\right)}^{4}+\frac{1.1\times 10^{8}V_{a}{}^{0.6}}{\varepsilon D^{0.4}}\left(T_{g}-T_{a}\right)\right]}^{\frac{1}{4}}-273$$

where T_g_ is the globe temperature (°C), T_a_ is the air temperature (°C), V_a_ is the wind speed (m/s), D is the globe diameter (set to 0.08m in this study), and ε is the emissivity of the black globe (set to 0.95).

In this study, PET index was used because it has been widely applied to analyze outdoor thermal comfort in various climates and it has the measurement unit (°C), which makes results easily understood by urban planners [21,26,45,46]. PET is based on the Munich Energy Balance Model for Individuals (MEMI) [47]. By definition, PET is the T_a_ at which, in a typical indoor room (T_mrt_ = T_a_; V_a_ = 0.1 m/s; water vapor pressure = 12 hPa), the heat balance of the human body (work metabolism 80W; heat resistance of clothing 0.9 clo) is maintained, with core and skin temperature equal to those under actual conditions [11]. PET is calculated by RayMan software, and the required meteorological variables for calculating include T_a_, T_mrt_, V_a_ and RH [48,49].

## 3. Results and discussion

### 3.1. Wind Speed Analysis

#### 3.1.1. Space Forms Effect on Wind Speed

Table 3 shows the average V_a_ of each space form in different seasons, and the value is the average V_a_ of three measurement points of each space form. It can be determined that in different seasons, the average V_a_ in the linear block, the square, and the enclosed block decrease in turn; the difference of V_a_ between the linear block and the enclosed block was relatively larger, between 0.87 and 1.41 m/s; the difference between the linear block and the square was smaller, at 0.45 m/s. This is because the venturi effect is more likely to occur in the linear block, where the airflow might accelerate, causing higher V_a_; the square is relatively open, where the airflow is less likely to be blocked by buildings, thus, the V_a_ was high but was lower than that of the linear block; while due to the strong blocking effect of the airflow by the buildings around it, the V_a_ in the enclosed block was the lowest.

Oke [50], Ali-Toudert et al. [25], and Jin et al. [10] all indicated that the V_a_ in the linear block increased as the angel of the prevailing wind direction and the axis of the linear block was reduced. In this study, the prevailing wind direction on the measured day in winter was west, which is close to parallel to the axis of the linear block. The average V_a_ was significantly higher than the measured days of the other two seasons. Although the prevailing wind direction (south) of the transition season was close to perpendicular to the linear block axis, due to the relatively high inflow V_a_ and complicated space configurations, the effect of airflow disturbance was enhanced so the average V_a_ in the linear block was still high. In addition, the average V_a_ in the square and the enclosed block was directly proportional to the inflow V_a_ and was only slightly affected by the prevailing wind direction.

#### 3.1.2. Space Configurations Effect on Wind Speed

**Figure 6:**
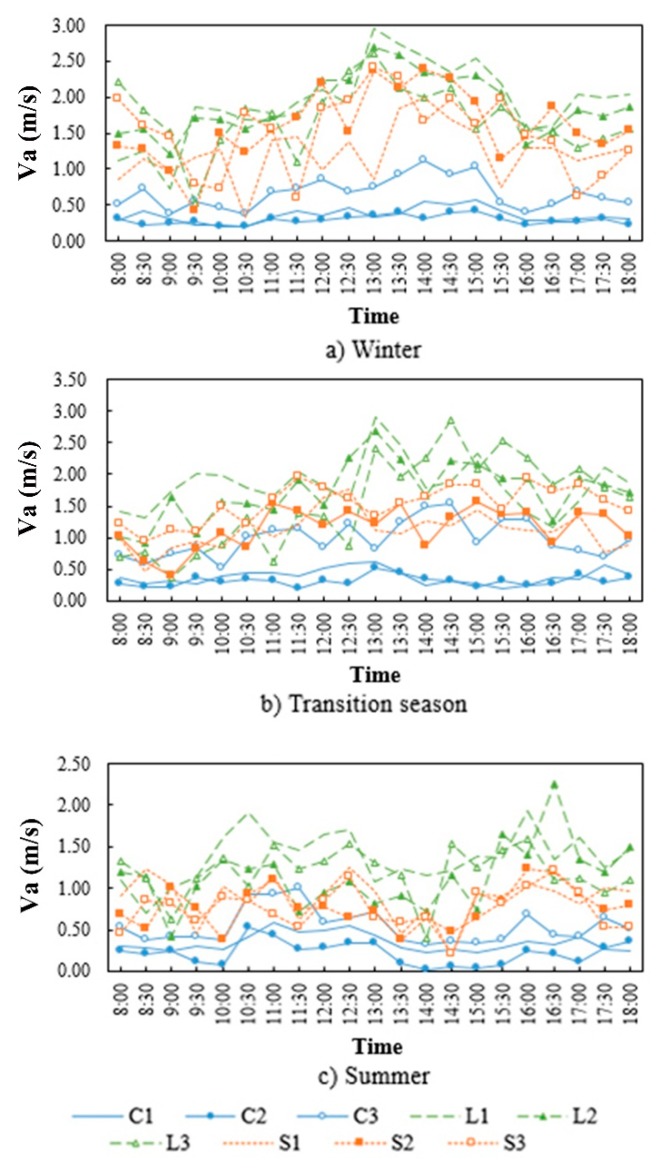
Temporal variation of wind speed for each measurement point in different seasons.

Figure 6 shows the temporal variation of V_a_ at each measurement point in different seasons. The variation range of V_a_ in different configurations of the linear block was larger, and smaller in the square, whereas the variation of V_a_ in the enclosed block was relatively mild. In addition, the differences of V_a_ between points were in direct proportion to the inflow V_a_, in the transition season with higher inflow V_a_, the maximum difference of V_a_ between points reached 4.1 m/s. 

Table 4 shows the standard deviation of V_a_ at each measurement point in different seasons. The standard deviations of V_a_ for the measurement points in the enclosed blocks followed a descending order: C3, C1, and C2. The fluctuation range of V_a_ in the semi-enclosed block was larger than in the full-enclosed block. Moreover, the higher the SVF of the full-enclosed block, the larger the fluctuation range of V_a_. In addition, the fluctuation range of V_a_ in the linear blocks and the squares did not show a clear tendency of change.

**Figure 7:**
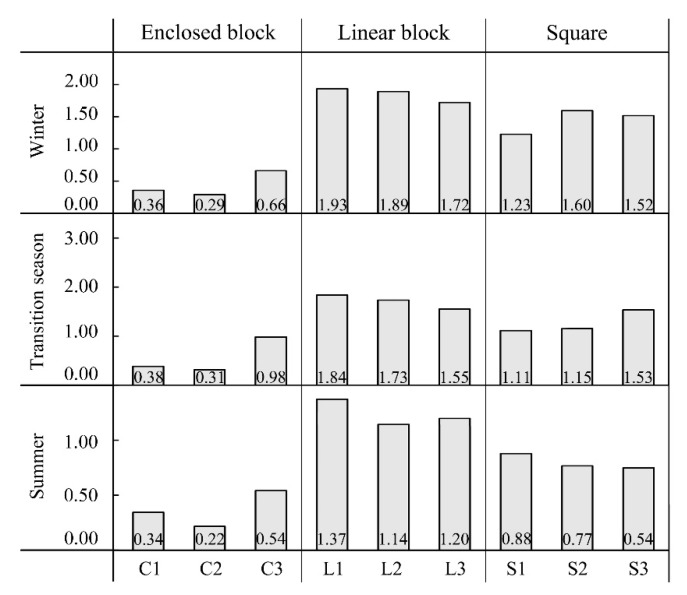
Average wind speed (m/s) of each measurement point in different seasons.

Figure 7 shows the average V_a_ of each measurement point in different seasons. By comparing the effects of different configurations of each space form on V_a_, it can be determined that in the linear block, when the inflow V_a_ was high (in the transition season and winter), although there was a big difference between the prevailing wind directions (close to perpendicular and parallel to the axis of the space respectively), the change tendencies of the average V_a_ of the measurement points were consistent where points L1, L2, and L3 decreased in order. When the inflow V_a_ was low (in summer), the average V_a_ of point L3 was slightly higher than that of point L2. Krüger et al. [51] have shown that when the axis of the linear block is parallel, perpendicular or oblique to the prevailing wind direction, the V_a_ is higher in spaces with a higher SVF (lower H/W ratio). The difference with the results in summer is due to the spatial configurations around the measurement points in this study being more complicated. When the V_a_ of the incoming flow was small, the effect on V_a_ distribution was high. In terms of the enclosed block, the average V_a_ of points C3, C1, and C2 decreased in order in all seasons. The V_a_ in the full-enclosed block was lower than that in the semi-enclosed block due to a higher extent of enclosing, therefore, the obstruction effect on the airflow was more remarkable. Moreover, the lower the SVF of the full-enclosed block, the stronger the obstruction effect and the smaller the V_a_. However, there was no significant tendency of change in the average V_a_ in squares with different space configurations in different seasons.

### 3.2. Air Temperature and Mean Radiation Temperature Analysis

#### 3.2.1. Space Forms Effect on Air Temperature and Mean Radiation Temperature

Table 5 and Table 6 show the average T_a_ and T_mrt_ of each space form in different seasons, respectively, and the values are the average T_a_ and T_mrt_ of three measurement points of each space form. In summer and the transition season, the average T_a_ and T_mrt_ of the square, the linear block and the enclosed block decreased in order, and compared with summer, the differences of the average T_a_ and T_mrt_ between the linear block and the enclosed block in the transition season were smaller at 0.2 °C and 6.8 °C respectively. In winter, the average T_a_ and T_mrt_ of the square, the enclosed block and the linear block decreased in order, the difference of the average T_a_ between square and the enclosed block was only 0.3 °C, and the average T_a_ and T_mrt_ in the enclosed block were, respectively, 0.9 °C and 1.0 °C higher than that in the linear block. This was because the maximum sun elevation angle decreased orderly in summer, the transition season and winter, the effects of solar radiation in the linear block and the enclosed block weakened, and the effects of the long-wave radiation heat of the building walls on T_a_ and T_mrt_ gradually strengthened. 

In addition, the enclosing extent of the enclosed block was high, thus the heat could not easily dissipate, resulting in a gradual decrease in the difference of the temperature between the two space forms. Moreover, in winter, the two space forms almost received no sun’s rays, so the temperature in the enclosed block was higher than that in the linear block due to the long-wave radiation. In addition, due to the longer time of direct solar radiation, the average T_a_ and T_mrt_ in the squares were relatively higher.

#### 3.2.2. Space Configurations Effect on Air Temperature and Mean Radiation Temperature

**Figure 8:**
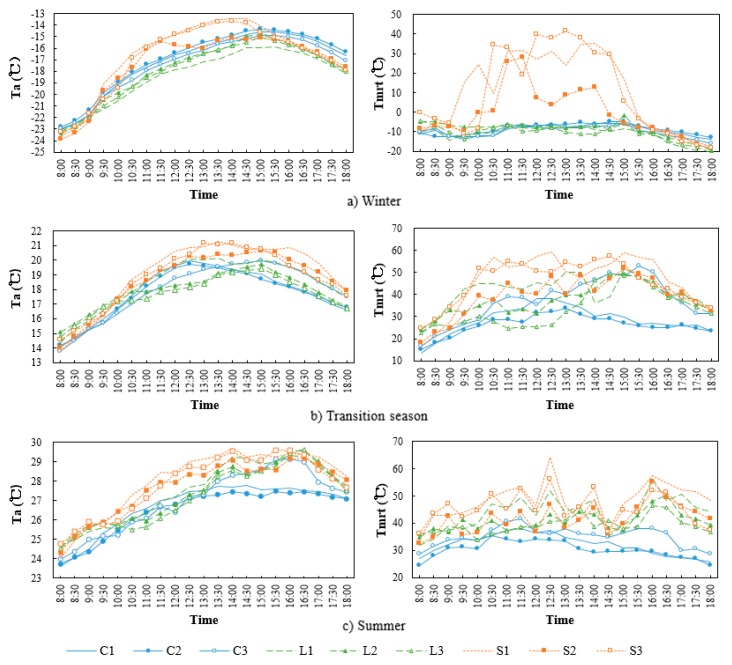
Temporal variation of air temperature and mean radiation temperature for each measurement point in different seasons.

Figure 8 shows the temporal variation of T_a_ and T_mrt_ at each measurement point in different seasons. The differences of T_a_ and T_mrt_ between the points increased as the sun elevation angle increased, the difference of temperature between the points began to increase since 9:30 in winter and the transition season, which began even earlier in summer. 

The maximum differences of T_a_ and T_mrt_ were, respectively, 2.2 °C (14:00) and 30.4 °C (12:30), both of which appeared between the square and the enclosed block. The maximum differences of T_a_ and T_mrt_ were, respectively, 3.4 °C (13:30) and 2.8 °C (13:00) in winter and in the transition season, both of which appeared between the square and the linear block. Evidently, the maximum differences between points in different seasons appeared between 12:30 and 14:00, and the T_a_ and T_mrt_ were significantly higher in the square. In addition, as the maximum sun elevation angle decreased orderly in summer, the transition season and winter, the maximum temperature difference between the points increased. This is because when the sun elevation is lower, the blocking effect of the buildings to the solar radiation is more significant in the linear block and the enclosed block but is less significant in the square, causing larger temperature differences.

In addition, the speed of temperature increasing and decreasing of the squares was significantly higher than that of the other two space forms in three seasons. The reason is that the square had a relatively wider sky exposure and received more solar radiation, the temperature increased with the increase of the sun elevation angle, but when the sun’s position became low, the heat dissipated easily and the temperature decreased rapidly. In terms of the linear block and the enclosed block, the fluctuation range of T_mrt_ in the linear block was significantly larger than that in the enclosed block in summer and the transition season due to the effects of solar radiation and V_a_. Moreover, in winter, since the sun’s highest position is low, the linear block and the enclosed block received little direct solar radiation so the fluctuation of T_a_ and T_mrt_ was relatively mild.

**Figure 9:**
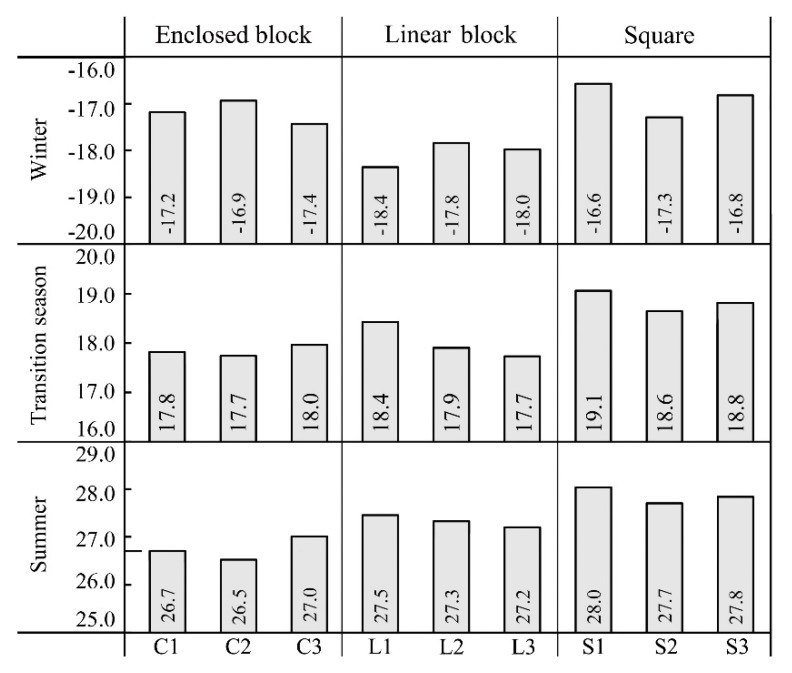
Average air temperature (°C) of each measurement point in different seasons.

**Figure 10:**
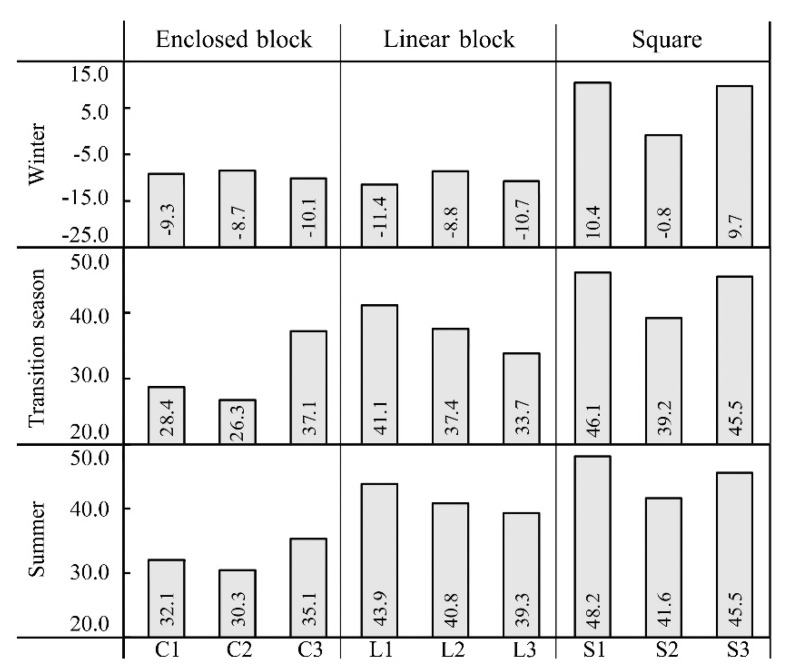
Average mean radiation temperature (°C) of each measurement point in different seasons.

Figure 9 and Figure 10 show the average T_a_ and T_mrt_ of each measurement point in different seasons, respectively. In terms of the squares, the average T_a_ and T_mrt_ of points S1, S3 and S2 decreased in order in different seasons; the differences of average T_mrt_ among the points were 0.6–2.7 °C; the average T_a_ of points S1 and S3 were similar, with a difference of about 0.2 °C. The average temperature of point S2 was low, the average T_a_ and T_mrt_ of point S2 were respectively about 0.4 °C and 6.8 °C lower than of point S1 both in the summer and transition season, and 0.7 °C and 11.2 °C lower in winter. Evidently, the temperature of squares was mainly affected by the south–north spacing and the building height on the south side. Yezioro et al. [32] indicated that the solar radiation can be effectively increased by extending rectangular urban squares in the regions of latitudes 26–34° in the following directions: N–S, NW–SE, and NE–S. However, extending in E–W is the most unfavourable for receiving solar radiation. The results of this study verified the above-mentioned viewpoint. The square which point S2 located had a small south–north spacing, and several high-rise buildings on the south side, leading to a large area of building shadow in the square, providing a lower temperature, which was more obvious when the sun‘s highest position was low in winter.

In terms of the linear block, the average T_a_ and T_mrt_ of points L1, L2 and L3 decreased in order both in summer and the transition season. The maximum differences of T_a_ and T_mrt_ among the points were 0.3 °C and 4.6 °C in summer and 0.7 °C and 7.4 °C in the transition season. This is because in the seasons with a relatively large sun elevation angle, the higher the SVF of the linear block, the more solar radiation is received, and the higher the temperature. In winter, the average T_a_ and T_mrt_ of points L2, L3 and L1 decreased in order, and the maximum differences of T_a_ and T_mrt_ were, respectively, 0.6 °C and 2.6 °C. It can be seen that the temperature in the linear block rose as the spacing between buildings increased in winter and was not directly related to the SVF of the space. Johansson [22] indicated a strong correlation between the T_a_ and T_mrt_ in the street and the SVF in summer and winter and the temperature significantly reduces when the SVF decreases. In this study, the results in winter differed from the previous study due to the differences of latitude and the urban texture of the studied location. Although the SVF of point L1 was larger than points L2 and L3, the spacing between buildings was narrower and the sun elevation was lower in Harbin in winter, so point L1 was always in the building shadow. In terms of points L2 and L3, there was a relatively wide spacing between buildings, therefore, the points could receive the sun’s rays coming from the east and the west for a short time in the morning and the afternoon so the average temperature was relatively higher and the received solar radiation was proportional to the spacing between buildings.

In terms of the enclosed block, the average T_a_ and T_mrt_ of points C3, C1 and C2 decreased in order both in summer and the transition season. Moreover, the maximum differences of T_a_ and T_mrt_ were respectively 0.5 °C and 4.8 °C in summer, and 0.3 °C and 10.8 °C in the transition season. This is because point C3 is located in a semi-enclosed block. Thus, when the sun’s elevation was low in the late afternoon, it was still exposed to the sun’s rays coming from the opening in the west, so its average T_a_ and T_mrt_ were the highest. In addition, C1 and C2 are located in the full-enclosed block, which can only receive the direct solar radiation when the sun elevation is relatively high. Moreover, the higher the SVF value, the more solar radiation can be received and the higher the temperature. In winter, the average T_a_ and T_mrt_ of points C2, C1, and C3 decreased in order, and the maximum differences of T_a_ and T_mrt_ were, respectively, 0.5 °C and 1.4 °C. Martinelli et al. [26] indicated that as the sun elevation angle is small in winter, and the effect of direct solar radiation on the courtyard space is weak, so the reduction of the SVF increases the reflect long-wave radiation of the buildings and reduces heat dissipation. However, the results are only suitable for the full-enclosed block. Point C3 is located in a semi-enclosed block. Although the SVF is lower, the smaller area of the building walls and faster heat dissipation led to a lower temperature. Therefore, the T_a_ and T_mrt_ of the full-enclosed block were higher than those of semi-enclosed block in winter, and the smaller the SVF of the full-enclosed block, the higher the temperature.

### 3.3. Thermal Comfort Analysis

#### 3.3.1. Space Forms Effect on PET

Table 7 shows the average PET value of each space form in different seasons, and the value is the average PET of three measurement points of each space form. In the transition season, the PET values of the square, the enclosed block and the linear block decreased in order and the average PET values in the square and the enclosed block was 4.6 °C and 1.1 °C higher than that in the linear block, respectively. This is because the T_mrt_ and V_a_ of the square were higher and lower than the linear block, respectively, the PET of the square was significantly higher. Although the T_mrt_ of the enclosed block was lower than that of the linear block, the V_a_ was significantly lower, so the PET of the enclosed block was slightly higher than that of the linear block. In winter, the PET values in the square, the enclosed block and the linear block decreased in order and the difference between the square and the enclosed block was only 0.9 °C due to the great different PET values in squares with different configurations. Lin et al. [52] pointed out that in winter, as the SVF decreases, thermal comfort conditions becomes low and the PET values of the square, the linear block (pass way) and the enclosed block (atrium) decrease in order. The results of this study differ from those because the measurement location was different. The latitude of Harbin is high, and the sun elevation angle in winter is small, as a result, the enclosed block and the linear block can receive little direct solar radiation, but as the enclose block is greatly affected by the long-wave radiation heat of the building walls and the V_a_ is lower, the PET value is slightly higher than that of the linear block; while the PET value of the square is more affected by the space configurations, in which the PET value of the square which S2 located in is lower than that of the enclosed block because of low T_mrt_ and relatively high V_a_. In summer, the average PET values of the square, the linear block and the enclosed block decreased in order and the average PET value of the square and the linear block was 5.4 °C and 2.3 °C higher than that of the enclosed block respectively, because the amount of solar radiation received increased significantly with the increase of SVF values in summer.

Lai et al. [53] pointed out in a study on outdoor thermal comfort in cold regions of northern China that the PET range of the “neutral” thermal sensation was 11–24 °C; moreover, “neutral” was perceived to be the most comfortable sensation in the transition season, “slightly cool” in the hot season, and “slightly warm” in the cold season. Thus, the thermal environments of all outdoor space forms are relatively comfortable in the transition season and less comfortable in winter and summer, so researchers should focus more on improving outdoor thermal comfort conditions in winter and summer.

#### 3.3.2. Space Configurations Effect on PET

**Figure 11:**
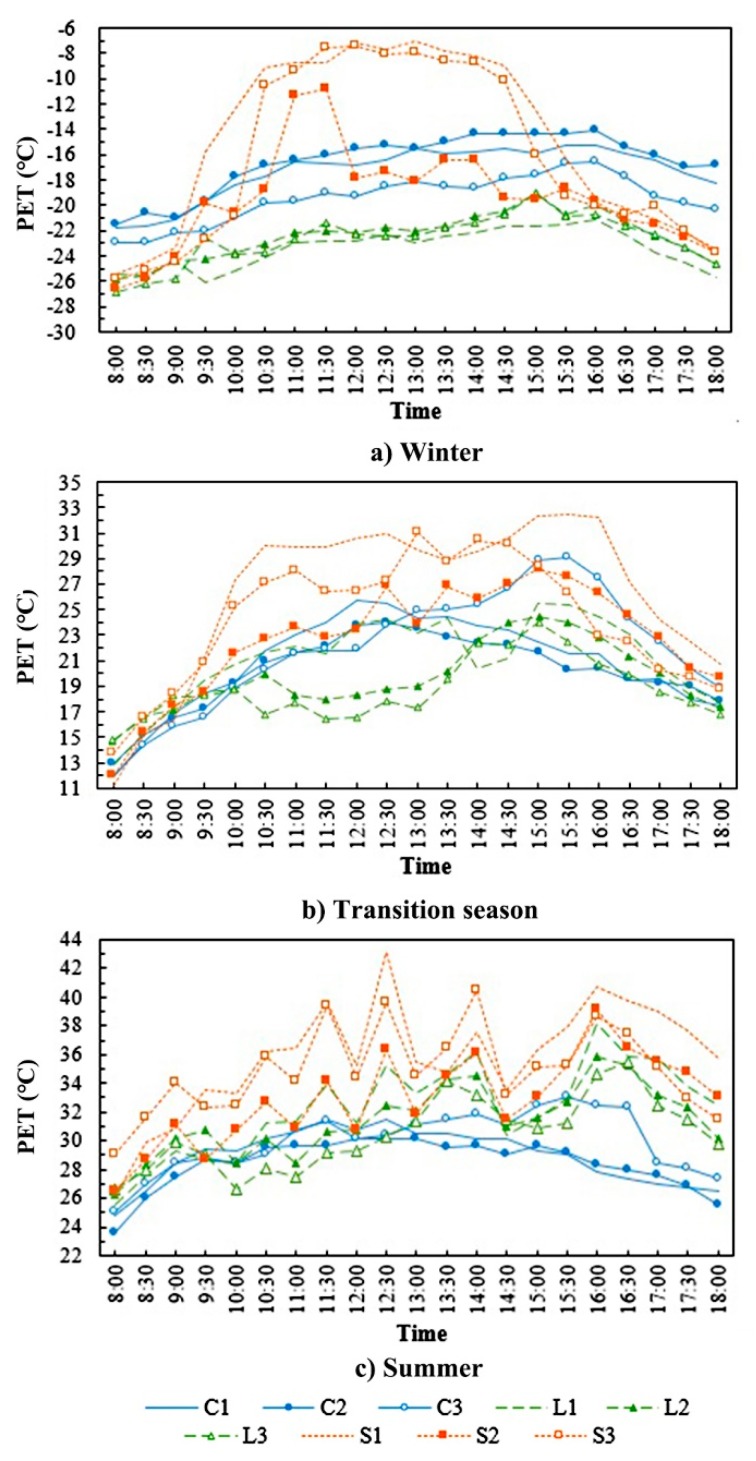
Temporal variation of PET for each measurement point in different seasons.

Figure 11 shows the temporal variation of PET at each measurement point in different seasons. It can be determined that the variation tendencies of PET of all points were basically consistent with the T_mrt_. This is because the T_mrt_ directly affected thermal comfort [11,44]. In addition, the differences between the measurement points increased as the solar radiation intensity rose, but the maximum differences in different seasons were close, all about 15.0 °C. The maximum difference appeared in summer (12:30), between the square and the enclosed block and appeared, respectively, at 12:00 and 13:00 in the transition season and in winter, both between the square and the linear block, showing that the thermal comfort conditions of different outdoor space forms during the same period of time differed greatly. In addition, due to the effects of solar radiation, the fluctuation range of the PET of the square was bigger than that of the linear block and the enclosed block. Particularly in winter, the PET of the square had a large diurnal swing and was 5–15 °C higher than that of the enclosed block and the linear block between 10:30 and 14:30, except for point S2, which is basically in the shadow of buildings. Besides, the PET in the enclosed block and the linear block were relatively stable and ranged from −27 to −14 °C. Moreover, the PET ranged from 12 to 32 °C and from 23 to 43 °C for all points between 8:00 and 18:00 in the transition season and summer, respectively.

**Figure 12:**
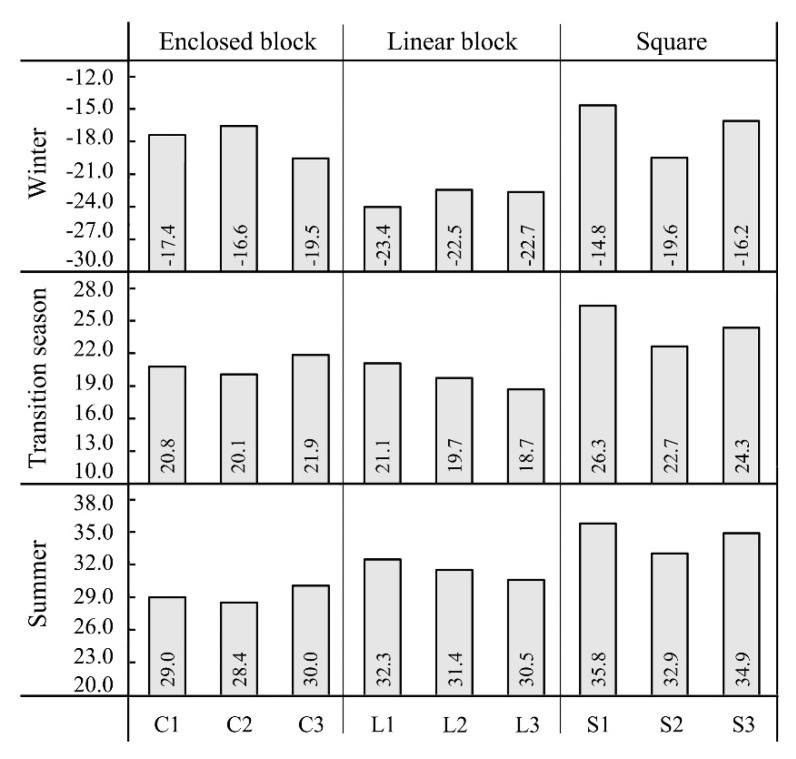
Average PET (°C) of each measurement point in different seasons.

Figure 12 shows the average PET value of each measurement point in different seasons. By comparing the effects of different configurations of each space form on thermal comfort conditions, it can be determined that for the squares, the average PET values of points S1, S3, and S2 decreased in order both in winter and summer, in which the average PET of point S1 in winter was, respectively, 1.4 °C and 4.8 °C higher than point S3 and point S2 and the average PET of S1 in summer was, respectively, 0.9 °C and 2. 9 °C higher than S3 and S2. Thus, the PET value decreased as the south–north spacing of the squares was reduced and the height of the buildings on the south side rose, which was even more significant in winter.

In terms of the linear block, in summer, the average PET values of points L1, L2 and L3 decreased in order, and the average PET of point L1 was, respectively, 0.9 °C and 1.8 °C higher than point L2 and point L3. Thus, in summer, with a large sun elevation angle, the amount of solar radiation received increased as the SVF increased, resulting in an increase in the PET value and a lower thermal comfort. In winter, the average PET values of points L2, L3, and L1 decreased in order, the average PET of point L2 was respectively 0.2 °C and 0.9 °C higher than L3 and L1. This is because in winter, with a small sun elevation angle, the amount of solar radiation received in the linear block is directly proportional to the spacing between buildings, so thermal comfort conditions will be improved as the spacing increases. In addition, in comparison with summer, the average PET value among measurement points was not very different in winter, with a maximum difference of only 0.9 °C, showing that the thermal comfort conditions in different linear blocks did not have great differences. 

In terms of the enclosed block, in summer, the average PET values of points C3, C1 and C2 decreased in order and the average PET of point C3 was, respectively, 1 °C and 1.6 °C higher than point C1 and point C2; in winter, the average PET values of points C2, C1 and C3 decreased in order and the average PET of point C2 was, respectively, 0.8 °C and 2.9 °C higher than point C1 and point C3. Thus, in winter and summer, the thermal comfort condition of the full-enclosed block was better than the semi-enclosed block, and as the SVF of full-enclosed block decreased, the thermal comfort condition improved.

## 4. Conclusions

Through field measurements, this study reveals the effects of forms and configurations of three typical outdoor spaces (the linear block, the enclosed block, and the square) on the thermal environments and thermal comfort conditions at the pedestrian level in three seasons (summer, the transition season and winter) in severe cold regions in China. The conclusions are as follows:

In different seasons, the average value and the fluctuation range of V_a_ in the linear block, the square and the enclose block decreased in order. Besides, in different seasons, the average values and the fluctuation ranges of T_mrt_ and T_a_ in the square and were all the largest. In summer and the transition season, the average T_mrt_ and T_a_ in the linear block were larger than in the enclosed block and the fluctuation range of the T_mrt_ in the linear block was relatively larger. In winter, the average T_mrt_ and T_a_ in the enclosed block were slightly higher than in the linear block and the fluctuation ranges of the two were both mild.

T_mrt_ and T_a_ are the main factors that affect the outdoor thermal comfort condition of residential areas in severe cold regions. In summer, the T_mrt_ and T_a_ of the enclosed block, the linear block and the square increased in order, while the outdoor thermal comfort of those decreased in order. In the transition season, the T_mrt_ and T_a_ of the square, the linear block and the enclosed block decreased in order, but the V_a_ of the enclosed block was significantly lower than that of the linear block, so the PET of the square, the enclosed block and the linear block decreased in order. In winter, the T_mrt_ and T_a_ of the linear block were lower, and V_a_ of that was higher compared with the other forms, so the outdoor thermal comfort of the linear block was the worst. Besides, the outdoor thermal comfort of the square and the enclosed block was greatly affected by the space configurations. In addition, the thermal environment in different space forms was relatively comfortable in the transition season, but was uncomfortable in winter and summer.

For the square, the T_mrt_ and T_a_ increased with the increase of south–north spacing and the decrease of building height on the south side, while the PET had the same trend. And the phenomenon was more significant in winter. For the linear block, in summer the T_mrt_ and T_a_ decreased as the SVF decreased, while the thermal comfort conditions were improved; in winter the T_mrt_ and T_a_ increased as the spacing between buildings increased, while the thermal comfort conditions were improved. However, in comparison with summer, the effects of different configurations of the linear block on the thermal comfort were not significant. For the enclosed block, the T_mrt_ and T_a_ in the semi-enclosed block were higher than in the full-enclosed block, and the temperature of the full-enclosed block was directly proportional to the SVF value in summer, whereas the relationships of them were opposite in winter. Moreover, the thermal comfort conditions of the full-enclosed block were better than those of the semi-enclosed block, and the thermal comfort conditions of the full-enclosed block were improved as the SVF value decreased in both winter and summer.

Currently, due to climate change and accelerated urbanization, not only the cold climate but also the hot climate in severe cold regions should be considered during residential areas planning. Therefore, the enclosed block is recommended because of providing a relatively comfortable microclimate in both winter and summer. Furthermore, the enclosed block should adopt the form of the full-enclosed, and on the premise of meeting the requirements of use, the SVF of the space should be reduced to improve outdoor thermal comfort. Besides, for squares, wide south–north spacing and low buildings on the south side should be adopted to improve the thermal comfort in winter, and deciduous trees can be planted on the south of the squares to reduce direct solar radiation, thus improving the thermal comfort in summer [54]. For the linear block, it is appropriate to set up high-rise residential buildings and widen the south–north spacing of buildings to a certain extent.

A limitation of this study is the examination of a specific orientation. The orientation of space forms has an important influence on outdoor thermal environment, especially on the T_mrt_ and V_a_ [25,55]. Therefore, the effects of different orientations on outdoor thermal comfort should be studied in future research. Besides, another parameter that plays an important role in the outdoor thermal comfort of residential areas is the green plant, and the green space planning needs to be based on the outdoor space form [56,57]. Our recommendation for future research on the residential areas of severe cold regions is to study the influences of different layout patterns of building and green plants on outdoor thermal comfort. In addition, more field measurements and numerical studies of different locations are needed to improve the reliability and universality of research results.

## Figures and Tables

**Table 1:** Instruments used for measurement of meteorological parameters.

Parameter	Model	Range	Accuracy
Air temperature	BES-02	−30–50 °C	±0.5 °C
Relative humidity	BES-02	0–99%	±3%
Globe temperature	BES-01	−30–50 °C	±0.5 °C
Wind speed	NK 4500	0.1–60 m/s	±0.1 m/s

**Table 2:** The meteorological data of the measured days in different seasons.

Season	Average T_a_	Average RH	Average V_a_	Maximum V_a_	Wind Direction
Winter	−21.2 °C	60%	2.45 m/s	4.7 m/s	West
Transition	12.2 °C	41%	3.8 m/s	7.1 m/s	South
Summer	24.5 °C	64%	1.15 m/s	3.7 m/s	Southeast

**Table 3:** Average wind speed (m/s) of each space form.

Season	Enclosed Block	Linear Block	Square
Winter	0.44	1.85	1.45
Transition season	0.56	1.71	1.27
Summer	0.37	1.24	0.80

**Table 4:** The standard deviation of wind speed (m/s) for each measurement point.

Season	C1	C2	C3	L1	L2	L3	S1	S2	S3
Winter	0.10	0.06	0.21	0.52	0.40	0.45	0.37	0.49	0.52
Transition season	0.11	0.08	0.28	0.39	0.44	0.71	0.28	0.32	0.29
Summer	0.11	0.13	0.21	0.30	0.39	0.29	0.26	0.23	0.24

**Table 5:** Average air temperature (°C) of each space form.

Season	Enclosed Block	Linear Block	Square
Winter	−17.2	−18.1	−16.9
Transition season	17.8	18.0	18.8
Summer	26.7	27.3	27.9

**Table 6:** Average mean radiation temperature (°C) of each space form.

Season	Enclosed Block	Linear Block	Square
Winter	−9.3	−10.3	6.4
Transition season	30.6	37.4	43.6
Summer	32.5	41.3	45.1

**Table 7:** Average PET (°C) of each space form.

Season	Enclosed Block	Linear Block	Square
Winter	−17.8	−22.9	−16.9
Transition season	20.9	19.8	24.4
Summer	29.1	31.4	34.5
